# Intratumoral TLR4 agonist therapy elicits antigen-specific T cell clonal expansion in metastatic leiomyosarcoma: a case series

**DOI:** 10.3389/fonc.2026.1829331

**Published:** 2026-05-20

**Authors:** Jessica S. Zhang, Juliana C. K. Ng, Pedro Hermida de Viveiros, Rusul Al-Marayaty, Nourhane Al Akoum, Himaly Shinglot, Weiqing Jing, Valeria Vizcaino, Borislav A. Alexiev, Lee D. Cranmer, Elizabeth T. Loggers, Hannah Abrams, Michael J. Wagner, Venu G. Pillarisetty, Edward Y. Kim, Robert H. Pierce, Peter Berglund, Robin L. Jones, Hailing Lu, Jan ter Meulen, Seth M. Pollack, Yongwoo David Seo

**Affiliations:** 1 Department of Medicine, Northwestern University Feinberg School of Medicine, Chicago, IL, United States; 2 Department of Pathology, Northwestern University Feinberg School of Medicine, Chicago, IL, United States; 3 Department of Medicine, City of Hope Cancer Center, Los Angeles, CA, United States; 4 Clinical Research Division, Fred Hutchinson Cancer Center, Seattle, WA, United States; 5 Sarcoma Center, Dana Farber Cancer Institute, Boston, MA, United States; 6 Department of Surgery, University of Washington, Seattle, WA, United States; 7 Department of Radiation Oncology, University of Washington, Seattle, WA, United States; 8 Attivare, Natick, MA, United States; 9 HDT Bio, Seattle, WA, United States; 10 Royal Marsden and Institute for Cancer Research, London, United Kingdom; 11 Pfizer, Bothell, WA, United States; 12 Obsidian Therapeutics, Cambridge, MA, United States; 13 Department of Surgical Oncology, Medical College of Wisconsin, Milwaukee, WI, United States

**Keywords:** Cancer vaccination, intratumoral injection therapy, metastatic leiomyosarcoma, NY-ESO-1, T cell clonal expansion, TLR4 agonist, tumor microenvironment

## Abstract

Metastatic soft tissue sarcomas (STSs) are a heterogeneous group of rare mesenchymal tumors whose treatment options are limited. Our group has been interested in utilizing the synthetic toll-like receptor 4 (TLR4) agonist, glycopyranosyl lipid A in stable-emulsion formulation (GLA-SE), in patients with advanced STSs. Here, we report a case series of three patients with metastatic leiomyosarcoma who had superficial lesions amenable to intratumoral (IT) injection. We explored the safety, tolerability, and immunologic effects of IT GLA-SE given at 20 μg weekly for 12 weeks, both alone and in combination with radiotherapy. In this small case series, treatment was well tolerated, with no serious adverse events observed. Clinically, although no patient had significant tumor shrinkage, two had notable stability in their target lesions despite systemic disease progression. Immunologically, IT GLA-SE induced a clonal expansion of T cells intratumorally in one patient and in peripheral blood in another, suggesting an antitumor effect both at the site of injection and systemically. Moreover, GLA-SE elicited clonal expansion of systemic T cells targeting a well-known immunogenic cancer antigen, NY-ESO-1. The augmented expression of T cells targeting NY-ESO-1 in the systemic circulation of our patient following IT-GLA-SE warrants further investigation, given the promising role of NY-ESO-1 in current and future T-cell based immunotherapies. Together, these findings demonstrate the potential of IT GLA-SE to elicit antigen-specific T cell clonal expansion as a component of immunotherapeutic strategies to combat metastatic STSs. Expanded studies are needed to explore the clinical and immunologic effects of IT GLA-SE, particularly in combination with other immunotherapies.

## Introduction

Soft tissue sarcomas (STSs) are a heterogeneous group of rare mesenchymal tumors composed of over 70 distinct histological subtypes, and together constitute roughly 1% of all cancers (1). Despite advances in treatment options, outcomes in patients with advanced disease remain poor, and the median overall survival for patients with metastatic STSs remains only 1.5–2 years (2, 3). Glycopyranosyl lipid A in stable-emulsion formulation (GLA-SE) is a synthetic toll-like receptor 4 (TLR4) agonist that has been used in numerous instances as a vaccine adjuvant (4, 5). Because of its immunologic effects, intratumoral (IT) injection of GLA-SE has been explored as an immunotherapeutic treatment in several tumor types (6, 7), and investigators have also examined the synergistic effects of TLR agonists combined with radiotherapy to stimulate anti-tumor immunity (8, 9).

We recently reported the results of our phase 1 nonrandomized pilot study exploring the safety and tolerability of IT GLA-SE injections in combination with radiotherapy for patients with advanced STSs. In our first two cohort (n=12), patients received weekly outpatient IT GLA-SE injections (at doses of 5 and 10 μg) in combination with palliative radiotherapy to their injected tumors. Treatment was well tolerated, with all patients achieving local control of their injected tumors after 8 doses. Immunologically, patients with durable local responses (n=5) showed signs of T cell receptor (TCR) clonal expansion in tumor-infiltrating lymphocytes (TILs) that were later detected in systemic circulation, suggesting a systemic antitumor effect (10). Moreover, the dominant clones that emerged were found to have expanded from numerous rearrangements coding for the same binding sequence, suggesting clonal convergence toward specific tumor antigens.

Following the above published cohorts, we describe here a case series of three additional patients treated as a third cohort within the same trial. These patients received weekly single-agent GLA-SE at a higher dose of 20 μg for 6 weeks, followed by the combination of GLA-SE injections with radiotherapy for an additional 6 weeks. Given the promising results from the first two cohorts who had received simultaneous IT GLA-SE and radiotherapy, this last cohort was launched to better elucidate the individual effects of IT GLA-SE and radiotherapy delivered sequentially, and to observe safety and tolerability of IT GLA-SE at a higher dose. To improve our understanding of the immunomodulatory effects of GLA-SE injections, we explored changes in the T cell receptor (TCR) repertoire, including changes in TCR clonality and diversity in tumor-infiltrating lymphocytes (TILs) and peripheral blood mononuclear cells (PBMCs), before treatment, after GLA-SE injections alone, and after GLA-SE injections with radiotherapy.

## Methods

### Study design and patients

This case series was treated within an amendment to an open-label, phase 1 nonrandomized controlled trial (NCT02180698) and was conducted at the Seattle Cancer Center Alliance and Fred Hutchinson Cancer Research Center between June 22, 2016, to August 28, 2018. This cohort was planned to include a total of eight patients, though only three patients were ultimately enrolled prior to trial closure due to funding reasons. The study protocol was approved by the Fred Hutchinson Cancer Research Center Institutional Review Board and was conducted in accordance with the International Conference on Harmonization Guidelines for Good Clinical Practice and the Code of Federal Regulations. All patients provided written informed consent.

Eligible patients were ≥18 years of age with metastatic STS with a superficial tumor requiring palliative radiation that was safely accessible for outpatient IT injection. Patients were required to have an Eastern Cooperative Oncology group (ECOG) performance status of 0–2 and adequate hematologic, renal, and hepatic function. Patients with major comorbidities, those on chronic immunosuppression, antibiotics for systemic infections, and systemic anticancer therapy less than two weeks prior were excluded.

### Treatment and endpoints

**Figure 1:**
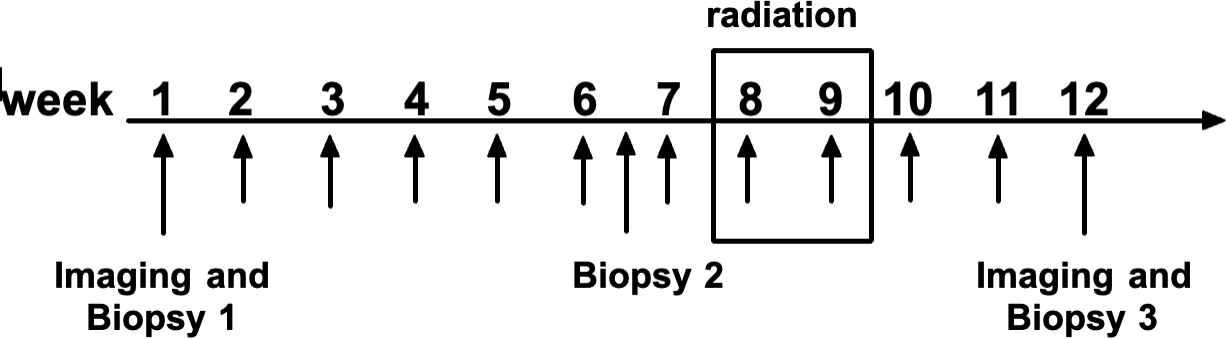
IT GLA-SE was given weekly for 12 weeks. Imaging and biopsies were taken at week 1, prior to first injection. Patients were rebiopsied after week 6, prior to radiation. Palliative radiation was then delivered to the targeted tumor at weeks 8 and 9. Imaging and biopsies were repeated again at week 12.

Patients received weekly IT GLA-SE injections (dose 20 μg) for 6 weeks, followed by palliative radiation to the target lesion and an additional 6 weeks of IT GLA-SE injections for a total of 12 weeks. Biopsies and imaging were done prior to the GLA-SE injections, at the 6-week mark before radiation, and after the full 12 weeks of injections (**Figure 1**). After treatment, imaging was continued every 6 weeks for 4 scans, then every 12 weeks after that, until progression or loss to follow-up.

We assessed safety and tolerability of IT GLA-SE injections with and without radiotherapy, graded using the Common Terminology Criteria for Adverse Events (CTCAE), version 5.0. Radiographic tumor responses in both the target lesion and distant tumor sites were assessed using Response Evaluation Criteria in Solid Tumors (RECIST), version 1.1. Immunologic effects were assessed via analyses of TCR repertoires of pre-, mid-, and post-treatment biopsy samples.

### Immunologic studies

Formalin-fixed paraffin-embedded (FFPE) tissue sample slides were stained with hematoxylin & eosin (H&E) and tumor presence was confirmed by pathologist review. Changes in TCR repertoires were analyzed via rapid expansion protocol of TILs (11), multiplex immunohistochemical analysis of core biopsy specimens (12), and TCR sequencing of rapid expansion protocol TILS and PBMCs (13). The immunoSEQ assay (Adaptive Biotechnologies) was used to sequence the TCR beta chain repertoires. TCR sequences from both TIL and PBMC samples obtained at pre-, mid-, and post-treatment intervals were analyzed using productive Simpson clonality, a metric used to quantify the expansion of specific TCR clones within each sample. Productive Simpson clonality was measured on a scale from 0-1, with values closer to 1 indicating a more monoclonal sample.

### Statistical analysis

Statistical analyses were conducted from August 2016 to November 2025 using Microsoft Excel and GraphPad Prism. Descriptive statistics were used to summarize demographic data, safety and tolerability results, local and systemic response, and exploratory immunologic response data. Local response was measured as the percent change in largest dimension of the target lesion, while systemic response was measured as percent change in the largest dimension of non-injected lesions.

## Results

### Patient characteristics

This case series includes three patients enrolled in the NCT02180698 trial at the 20 μg level. Demographic and clinical characteristics of each patient can be found in **Table 1**.The mean age was 65 (range 55-75) years, all had leiomyosarcoma, and all had superficial, palpable lesions amenable to outpatient injection. Patient 1 was a 75-year-old female who received 2 prior lines of systemic therapy. Her target lesion was a large 16 by 20 cm abdominal wall tumor. Patient 2 was a 66-year-old male who received 1 prior line of systemic therapy, and whose target lesion was a 3.4 x 3.0 cm lesion on his right arm. Patient 3 was a 55-year-old male who received 5 prior lines of systemic therapy, and whose target lesion was a 3.1 x 3.1 cm paraspinal mass.

**Table 1:** Baseline characteristics in 3 patients treated with IT GLA-SE injections followed by radiotherapy.

Patient number	Age	Sex	Sarcoma subtype	Site of IT GLA-SE injection	Size of injected lesion (cm)	Sites of disease	Number of prior lines of therapy
1	75	F	Leiomyosarcoma (uterine)	abdominal wall	16 x 20	soft tissue, uterine, pelvic, and abdominal wall	2
2	66	M	Leiomyosarcoma (non-uterine)	right arm	3.4 x 3.0	soft tissue, bone, and muscle	1
3	55	M	Leiomyosarcoma (non-uterine)	paraspinal	3.1 x 3.1	lung, stomach, small intestine, soft tissue, muscle, and abdominal wall	5

IT, intratumoral; GLA-SE, glycopyranosyl lipid A in stable-emulsion formulation.

### Safety and tolerability

Patient 1 died before completion of the study, after transitioning to hospice. She completed 6 weeks of IT GLA-SE injections followed by radiation to her abdominal wall mass (3,000 cGy in 6 fractions). She experienced no adverse events related to her treatment. Patients 2 and 3 completed 12 weeks of IT GLA-SE injections and radiation to their target lesions at the 6-week mark (3,000 cGy and 2,500 cGy, respectively) and tolerated treatment well. Patient 3 experienced several grade 1 adverse reactions, including fatigue and injection site reactions, but neither patient experienced any grade 2 or 3 adverse events, and no dose delays were required.

### Response results

Response results were only available for Patients 2 and 3, as Patient 1 transitioned to hospice shortly after her mid-treatment biopsy. Patient 2 was followed up for a total of 441 days, while Patient 3 was followed up for 125 days. Patient 2 had a 5.9% reduction in his target lesion (largest dimension 3.4 cm to 3.2 cm) after 6 weeks of injections, prior to radiation. The lesion was stable after receiving radiation and for the duration of his follow up. Systemically, Patient 2 had progression of disease at his 12-week imaging. Patient 3 had no change in his 3.1 cm target lesion throughout follow up. Systemically, Patient 3 showed progression of disease at his 12-week imaging. Neither patient demonstrated a systemic tumor response and neither experienced an additional reduction in size of their target lesions following palliative radiation.

### The impact of IT GLA-SE on the tumor microenvironment

**Figure 2:**
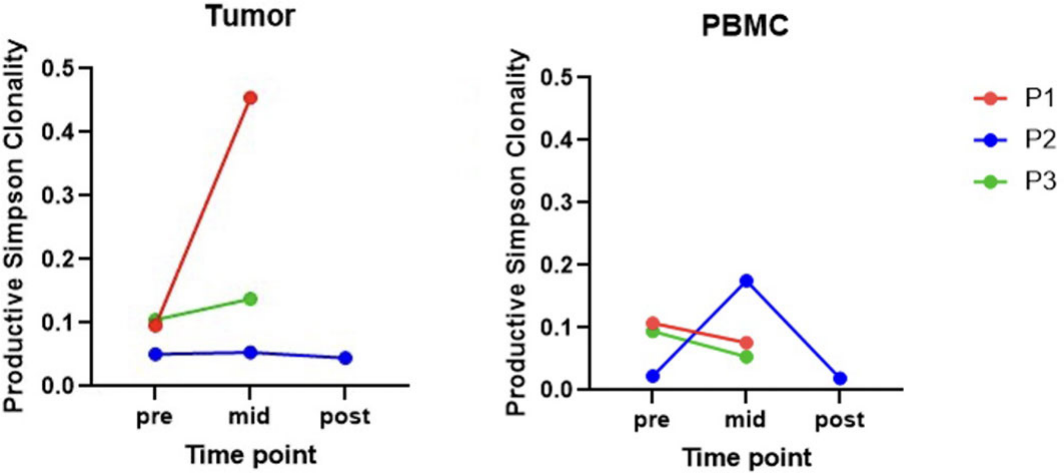
TIL; Tumor-infiltrating lymphocyte. PBMC; peripheral blood mononuclear cell. Productive Simpson clonality ranges from 0-1, where 1 represents a completely monoclonal sample. P1 and P3 do not have post-treatment data. P1’s TIL clonality increased from 0.10 to 0.45 between her pre- and mid-treatment biopsies. P2’s PBMC clonality increased from 0.02 to 0.18 between pre- and mid-treatment biopsies, then decreased back to 0.02 by the post-treatment biopsy.

**Figure 3:**
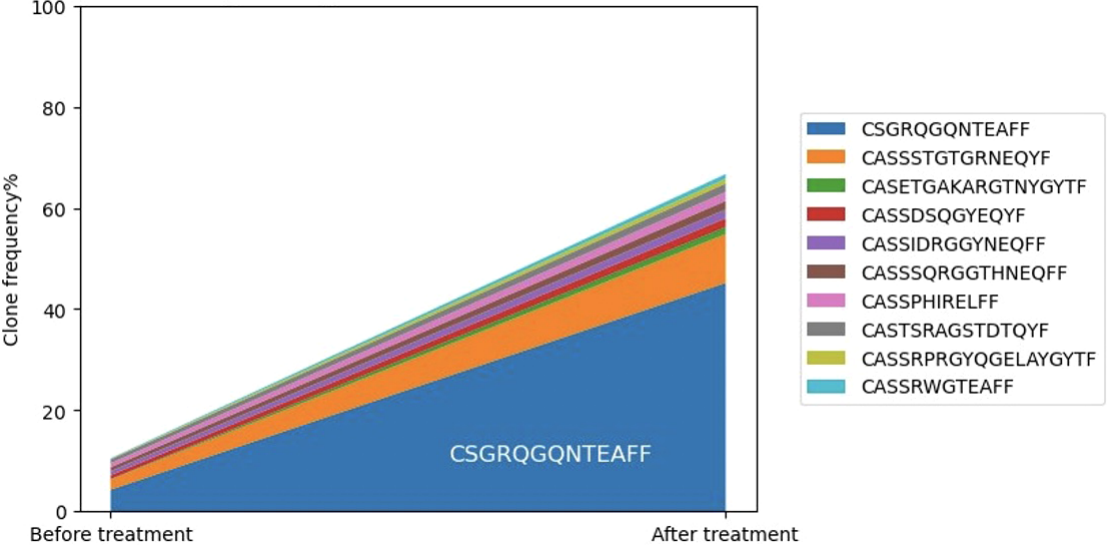
TIL; Tumor-infiltrating lymphocyte. In Patient 1, the dominant clone, CSGRQGQNTEAFF, expanded from a relative frequency of only 4% of all clonotypes prior to treatment to 45% of all clonotypes after 6 weeks of IT GLA-SE.

As an exploratory endpoint, multiplex immunohistochemical analysis was performed on pre-, mid- and post-treatment samples. In Patient 1, both CD4^+^ and CD8^+^ T cell infiltration increased (from 3.4 to 13.7% and 1.2 to 6.4%, respectively) at her mid-point biopsy. TCR sequencing was used to discern whether this increased T cell infiltration represented a generalized inflammatory response versus a clonal expansion against tumor antigens stimulated by the IT GLA-SE injections. TIL clonality in Patient 1 increased from 0.10 to 0.45 between her pre- and mid-treatment biopsies, after receiving 6 weeks of IT GLA-SE alone (**Figure 2**). The most prevalent clonotype, defined by a unique TCR amino acid sequence, expanded from pre- to mid-treatment samples, from a relative frequency of only 4% to 45% of all clonotypes. The rise of this dominant TCR clonotype, composed of the amino acid sequence CSGRQGQNTEAFF, suggests a clonal expansion of pre-existing T cells in response to the IT GLA-SE (**Figure 3**).

In contrast, neither Patient 2 nor 3 were found to have a significant increase in CD4^+^ or CD8^+^ T cell infiltration between their pre-treatment and mid-treatment biopsies. There was likewise no significant increase in TIL clonality observed from TCR sequencing (**Figure 2**). Patient 2, the only patient to receive a post-treatment biopsy, likewise saw no significant change in CD4^+^ and CD8^+^ T cell infiltration or TIL clonality between his mid-treatment and post-treatment biopsies.

### The impact of IT GLA-SE on circulatory T cells

Next, we explored whether IT GLA-SE was associated with changes in the TCR repertoires of circulatory T cells from PBMC samples that might suggest a systemic treatment effect. In Patient 1, who demonstrated a large increase in TIL clonality between pre- and mid-treatment biopsy samples, there was no apparent change in PBMC clonality with treatment (**Figure 2**). Interestingly, Patient 1’s dominant clone found in her mid-treatment TIL samples, CSGRQGQNTEAFF, was found to be present in both her pre-treatment and mid-treatment PBMC samples at very low frequencies (<0.01% frequency). However, the frequency of this clone in circulation did not appear to change at the mid-treatment point, following IT GLA-SE alone. Similarly, Patient 3 did not have a notable change in his PBMC clonality between pre- and mid-treatment samples.

**Figure 4:**
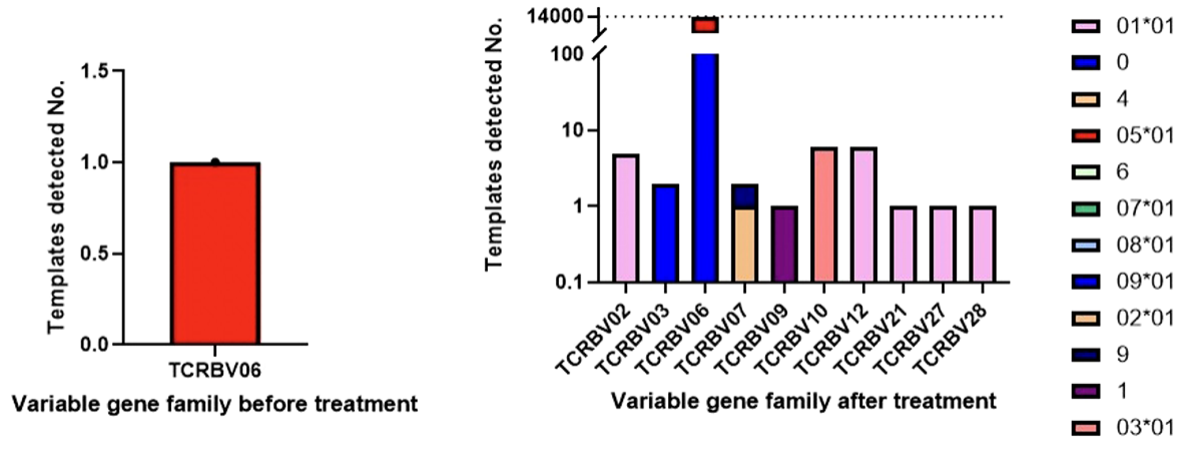
In Patient 2, the dominant TCR clonotype, CASSYVGNTGELFF, increased from a frequency of less than 0.01% in only 1 unique DNA rearrangement (1 template read) to 27% in mid-treatment samples, contributing 15,350 templates with 54 unique rearrangements from 10 different variable gene families, suggesting clonal convergence.

In Patient 2, there did appear to be an increase in PBMC clonality between pre- and mid-treatment samples, with clonality increasing from 0.02 to 0.18, although this increase was not sustained by the post-treatment biopsy (clonality 0.02). One TCR clonotype, CASSYVGNTGELFF, present in pre-treatment samples at a very low frequency of < 0.01%, expanded in mid-treatment samples to 27%. This clonotype, CASSYVGNTGELFF, became the dominant clone in the sample, contributing 15,350 templates with 54 unique rearrangements from 10 different variable gene families, suggesting clonal convergence (**Figure 4**). This dominant clonotype, CASSYVGNTGELFF, was not present in any of the TIL TCR sequences, though it was present in two of Patient 1’s pre-treatment PBMC samples (frequencies of 0.02% and 11% respectively).

## Discussion

This case series describes three patients with advanced leiomyosarcoma treated with IT GLA-SE injections, alone and in combination with radiotherapy. In this small cohort, treatment was safe, well-tolerated, and feasible in the outpatient setting. IT GLA-SE injections, with and without radiotherapy, did not lead to significant tumor shrinkage, though there did appear to be stability of the target lesions despite systemic progression. Such findings, along with those of our previous two cohorts, suggest that IT GLA-SE injection in combination with radiotherapy may be an effective strategy to control difficult and symptomatic tumors in patients with advanced disease. This cohort also extends the findings of our prior study by supporting safety at increased IT GLA-SE dose ranges. Nonetheless, this study has important limitations. Given the small sample size, our findings here, particularly regarding tumor response and immunologic response, are largely exploratory and hypothesis-generating. Our data is purely descriptive, and our results have not been powered to detect a clinical effect. Moreover, our lack of complete response data, particularly in Patient 1, likewise limits further generalizability of our findings. Our exploratory immunologic studies, rather, serve to provide insights into the effects of IT GLA-SE on both the tumor microenvironment and circulating T cells that may inform future immunotherapies for leiomyosarcomas.

The concept of “*in situ* cancer vaccination” has garnered significant attention as an immunotherapeutic strategy to combat cancer. By stimulating hosts’ immune systems to target tumor antigens, IT GLA-SE injection can potentially induce a significant anti-tumor effect, particularly when used synergistically with strategies like radiotherapy. In STSs, Su et al. demonstrated that the combination of radiation therapy and an IT TLR9 agonist delayed tumor growth and enhanced radiation response in autochthonous murine models (14). Mechanistically, they found that tumor response was mediated by the expansion of clonal intratumoral CD8+ T cells. Others have found intratumoral TLR4 agonist injection to be effective in stimulating tumor regression in orthotopic murine models with osteosarcoma (15). In fact, Cascini et al. found that intratumoral TLR9 agonist injection impaired growth of both the treated tumor and contralateral untreated tumors in murine models, demonstrating an abscopal effect (16). In human studies, combined IT TLR9 injection and radiotherapy induced both local and systemic response in patients with lymphoma (9). Similarly in patients with Merkel cell carcinoma, neoadjuvant IT GLA-SE was found to induce tumor regression when combined with radiotherapy and surgery (6). Mechanistically, IT GLA-SE was shown to have important immunostimulatory effects, including the induction of immune response-related genes, the colocalization of CD4^+^ and CD8^+^ T cells and macrophages in the TME, and increased number of unique clones both within the tumor and in the circulation that together, create a more favorable inflamed TME (6).

Here, we demonstrate that IT GLA-SE alone, prior to exposure to radiotherapy, was capable of inducing a local clonal expansion of discrete TIL TCRs, as seen in Patient 1. Notably, we also found that in Patient 2, TCR clonality expanded in PBMCs after IT GLA-SE alone, though this was not sustained by the post-treatment biopsy. The dominant clone in this case, CASSYVGNTGELFF, was found to be composed of many individual gene rearranged clones, suggesting clonal convergence towards a target tumor antigen. This same phenomenon was notably also found in the previously published cohort of this study, wherein dominant clones emerged at the amino acid level that were composed of very diverse TCR rearrangements at the gene level, suggesting a powerful antigen-specific T cell clonal response.

Moreover, the dominant clone found in the PBMC sample from Patient 2 was surprisingly also found in the pre-treatment PBMC samples from Patient 1. Given the rarity of two patients having a shared amino acid sequence of the complementarity-determining region 3 (CDR3) of the TCR, this particular sequence (CASSYVGNTGELFF) was investigated further. On literature review, CASSYVGNTGELFF has been identified as a TCR CDR3β sequence found to recognize an important cancer epitope, NY-ESO-1, a cancer-testis antigen that is rarely expressed in normal somatic tissues, but is aberrantly expressed in many solid tumors (17, 18). NY-ESO-1 has been shown to be heavily expressed in several STS subtypes (19, 20), making it an attractive target for T cell-based immunotherapies (21). NY-ESO-1 has already been successfully targeted in a number of studies on solid tumors such as metastatic melanoma and synovial cell sarcoma, including both as a vaccine and as adoptive T cell therapy (22–24). Interestingly, NY-ESO-1 expression has historically been low in both uterine and non-uterine leiomyosarcomas (25, 26), making the augmented expression of T cells recognizing NY-ESO-1 in our patient here unique. Unfortunately, immunohistochemistry was not done to corroborate the TCR findings here with actual NY-ESO-1 tissue expression, and is an important limitation to our findings. Future studies correlating immunologic effects of IT GLA-SE with immunohistochemical staining for NY-ESO-1 on biopsied tissue are warranted. Should IT GLA-SE be capable of augmenting expression of T cells targeting NY-ESO-1, IT GLA-SE could be a means of stimulating antitumor T cells for future T-cell based therapies. Moreover, IT GLA-SE may be useful in sensitizing sarcoma subtypes with traditionally low NY-ESO expression, such as non-uterine leiomyosarcomas, to emerging NY-ESO-1 targeted therapies, and future studies evaluating this hypothesis are warranted (25). Lastly, although the dominant intratumoral clonotype identified in Patient 1 (CSGRQGQNTEAFF) demonstrated striking clonal expansion, its target antigen remains uncharacterized, and whether it recognizes a defined tumor epitope as seen with CASSYVGNTGELFF, warrants future investigation.

Overall, the findings of this exploratory pilot study suggest IT GLA-SE is capable of inducing both local and systemic clonal expansion of anti-tumor T cells in patients with metastatic leiomyosarcomas. The ability of IT GLA-SE to stimulate a more favorable inflammatory state has important therapeutic implications for metastatic STSs, particularly in the wake of immune checkpoint inhibitors, which have shown activity in inflamed STSs. One recent trial (PEMBROSARC) evaluating the combination of IT TLR4 injection with pembrolizumab in patients with STS, found no clinical systemic response, but did not include radiotherapy (27). In the future, studies combining IT GLA-SE, radiotherapy, and immune checkpoint inhibitors in metastatic STSs may shed additional light on the immunomodulatory effects of IT GLA-SE. Moreover, IT GLA-SE and its ability to stimulate T cell recognition of the immunogenic cancer epitope NY-ESO-1 may have important implications for future adoptive T cell therapies, and also warrants further investigation.

## Data Availability

The raw data supporting the conclusions of this article will be made available by the authors, without undue reservation.
